# Validation of single measurement of 12-hour urine excretion for estimation of sodium and potassium intake. A longitudinal study

**DOI:** 10.1590/1516-3180.2017.0210031117

**Published:** 2018-04-16

**Authors:** Maria del Carmen Bisi Molina, Taísa Sabrina Silva Pereira, Aline Silva Porto, Raiane Pereira Silva, Nathália Miguel Teixeira Santana, Nágela Valadão Cade, José Geraldo Mill

**Affiliations:** I MSc, PhD. Associate Professor, Department of Integrated Health Education, Universidade Federal do Espírito Santo (UFES), Vitória (ES), Brazil.; II MSc. Doctoral Student of Public Health, Universidade Federal do Espírito Santo (UFES), Vitória (ES), Brazil.; III BSc. Master’s Student, Nutrition and Health Program, Universidade Federal do Espírito Santo (UFES), Vitória (ES), Brazil.; IV BSc. Nutritionist, Universidade Federal do Espírito Santo (UFES), Vitória (ES), Brazil.; V BSc. Nutritionist, Instituto Federal de São Paulo (IFSP), São Paulo (SP), Brazil.; VI MSc, PhD. Associate Professor, Department of Nursing, Universidade Federal do Espírito Santo (UFES), Vitória (ES), Brazil.; VII MD, PhD. Titular Professor, Department of Physiology, Universidade Federal do Espírito Santo (UFES), Vitória (ES), Brazil.

**Keywords:** Electrolytes, Diet, Urine specimen collection, Validation studies

## Abstract

**BACKGROUND:**

Evaluation of sodium and potassium intake can be carried out using different methods. Biological markers are able to capture intra and inter-individual variability and are used as separate measurements of consumption. The aim of this study was to test the validity of a single measurement of urinary sodium and potassium excretion as representative of habitual intake.

**DESIGN AND SETTING:**

Longitudinal study, federal university.

**METHODS:**

Food consumption data from a sample of adult university students and public servants (25 to 74 years old) were collected through 24-hour records and 12-hour urinary sodium and potassium excretion at five different times over a one-year period. The dietary data were entered into a nutritional research data software system and the sodium and potassium intakes were estimated. The variables were tested for normal distribution using the Kolmogorov-Smirnov test. One-way analysis of variance or the Kruskal-Wallis test was used to evaluate means. Correlations between measurements using Pearson or Spearman coefficients were calculated. The degree of agreement between the five measurements was given by the intraclass correlation coefficient.

**RESULTS:**

Satisfactory agreement was found between the five measurements of urinary sodium and potassium excretion over a year, with little variability in consumption.

**CONCLUSION:**

A single measurement of urinary sodium and potassium accurately estimated the usual average consumption of these electrolytes. This can be used in population-based studies.

## INTRODUCTION

One of the great challenges of nutritional epidemiology is to accurately determine the sodium and potassium intake in individuals’ diets.^1^ The dietary methods used to evaluate these nutrients may present different biases arising both from the information provided by individuals (through memory) and from the portion sizes and analytical instruments used in translating this nutrient information.^2^

Specifically, evaluation of sodium intake is complex due to the great variability within and between individuals.^3^ However, this situation is not identified in dietary methods for evaluating intake, since the amounts of ingredients in recipes, including salt, are standardized.^4^ The highest amounts of dietary sodium come from manufactured foods,^5^ which have known compositions that are the same for all consumers of that product, with little variation between different brands. Nonetheless, if a significant portion of the sodium intake comes from salt that is added during food preparation or through use of manufactured seasonings, it becomes more difficult to use dietary methods to accurately identify the amounts of this nutrient and its variability among individuals.

For this reason, 24-hour urinary excretion has been used as a marker of daily sodium intake, since under normal conditions 95% of what is ingested is eliminated through urine.^6^ Although not subject to the errors mentioned in relation to dietary methods, 24-hour urinary collection requires greater adherence by the individuals involved and presents higher costs. Considering the difficulties of collecting urine for 24 hours, 12-hour urine collection overnight has now been validated and has been used to estimate the daily sodium and potassium intake.^7^ However, given the great variability of sodium intake between different days, a single measurement may represent a limiting factor for the use of this method.

Although questionnaires, records or reminders are easier to use, they make it impossible to identify the variability among individuals in relation to addition of salt and seasonings in food preparation. This is minimized through measurement of urinary excretion.

The objective of the present study was to test the validity of a single measurement of urinary sodium and potassium excretion as representative of habitual intake. Thus, we evaluated the variability of the measurements throughout the year, while the participants’ usual diet did not change.

## METHODS

A longitudinal study was conducted on a sample of university students and public servants approached in a university, comprising adults (25 to 74 years old) of both sexes. Sociodemographic, anthropometric and hemodynamic data were collected, as well as data on dietary intake and urinary sodium and potassium excretion, at five different time points during a one-year period. All the procedures were carried out in the Cardiovascular Research Clinic of the Health Sciences Center of the Federal University of Espírito Santo (Universidade Federal do Espírito Santo, UFES). Before data collection was started, a pilot study on 10 people was carried out in order to calibrate the interviewers and researchers.

To calculate the sample size, we made assumptions of a correlation coefficient of approximately 0.6, statistical power of 80% and significance level of 5%. Thus, the sample size was established as 100 individuals. Nonetheless, we took into account the possibility that participants might be lost because of the requirement that 12-hour urine collections would need to be performed five times over a 12-month period. Therefore, 164 individuals were invited to participate.

Individuals with renal disease who were undergoing dialysis, pregnant women and individuals with cognitive limitations were excluded. Participants whose volumes of 12-hour urine collection were lower than 250 ml were excluded from the analysis.

### Data collection

Eligible participants at the university were approached and informed about the study and invited to participate. Soon after they had signed an informed consent statement, instructions were given and the material for the first urine collection was delivered. At that time, the participants were also informed of the timetable for the urine collection and food registration over the year. They also received a photographic album containing full-size photos of utensils to enable estimation of the sizes of the portions consumed, for the 24-hour food registration.

### Urine collection and anthropometric evaluation

Urine collection was performed on five occasions, with three-month intervals between them. On the first and last occasions, urine was collected over a 24-hour period, divided into two periods of 12 hours: daytime (from 7 am to 7 pm) and nighttime (from 7 pm to 7 am the following morning). On the other occasions, only a 12-hour urine collection was made, at night.

The urine was collected at home by the participants in sterile, previously labeled bottles after verbal and written instructions had been given regarding the collection times and storage conditions (in the refrigerator) to be observed for the urine bottles. For the analyses, we only used 12-hour night urine.

On the day when the participants handed over the first urine sample and food registry forms at the cardiovascular clinic, they underwent anthropometric and hemodynamic evaluations, in accordance with standardized techniques. A structured questionnaire was applied in order to investigate their use of medications and dietary supplements, along with their lifestyle habits. Weight was measured at the baseline and at the end of the study.

After the urine samples had been handed over, the volumes were measured in a graduated cylinder (10 ml) and all the aliquots were sent to the university laboratory to determine the sodium and potassium concentrations by means of the selective electrode method. The total amount of electrolyte excretion over the 12-hour periods was determined by multiplying the concentration by the volume of urine collected.

### 24-hour food registration

The nutrient intake was obtained by means of a 24-hour food register on all five occasions on which urine was collected. The participants were encouraged to record in detail all food and beverages consumed over the 24-hour period, with the aid of an album containing full-size photos of utensils, in order to estimate the sizes of the portions/volumes consumed. Data on the food register were checked by the researchers at the time when the forms were delivered.

The nutritional composition of the food items reported in the food register was estimated through identifying these items in the database of the Nutrition Data System for Research (NDSR) of the University of Minnesota.^8^ In addition, the Brazilian Table of Food Composition (TACO),^9^ of the State University of Campinas (Universidade Estadual de Campinas, UNICAMP), was used to identify the following foods: “*farinha de mandioca”* (cassava flour), *“farofa”*, *“pirão”*, *“dobradinha”, “cajá”* and *“açaí com guaraná”* (açaí pulp with guaraná syrup).

The nutritional composition of local preparations was calculated based on the individual components of each preparation, according to information in technical publications from teaching and research institutions. For each 100 grams of edible parts of the foods and preparations, the values of total energy (kcal), sodium (mg) and potassium (mg) were calculated. Energy adjustments were performed using the residual method, to correct the estimates for sodium and potassium intake.^10^

### Variables studied

The following data were collected and analyzed: sex, age (years), self-reported race/color (black, brown, white, indigenous and yellow), schooling, socioeconomic class (evaluated according to the Brazilian economic classification criteria, proposed by the Brazilian Association of Market Research Companies^11^), weight (kg), height (cm), energy (kcal), sodium and potassium from the dietary record (g) and urinary excretion of sodium and potassium (g).

Height was measured using a wall-mounted stadiometer (Seca, model 2161814009) with an accuracy of 1 mm. Individuals needed to be in a standing position, barefoot, looking straight ahead. Height measurements were made during the inspiratory period of the respiratory cycle. Body weight was measured with the subject still barefoot, on an electronic scale (Toledo, model 2096PP), with a capacity of 200 kg and a precision of 50 g. The body mass index (BMI) was calculated from body weight (in kg) divided by height in meters squared (m^2^).

### Statistical analysis

The normality of distribution of the continuous variables was determined by the by means of the Kolmogorov-Smirnov test. Pairs of means were compared using Student’s t test and comparisons between more than two means were made using one-way analysis of variance (ANOVA). Continuous variables were correlated by means of the Pearson and Spearman coefficients in cases of symmetrical and asymmetrical distribution, respectively. The strength of the association was considered null for r < 0.25, weak for 0.25 ≤ r < 0.5, moderate for 0.5 ≤ r < 0.75 or strong for r ≥ 0.75.^2^

The degree of agreement between the five measurements over the course of the year was given by means of the intraclass correlation coefficient, also called the reproducibility coefficient (RC). The intraclass correlation coefficient estimates the fraction of the total variability of measurements that is due to variations between individuals. For the intraclass correlation coefficient assessment, the following classification was used: weak, intraclass correlation coefficient < 0.4; satisfactory, 0.4 ≤ intraclass correlation coefficient < 0.75; and excellent, intraclass correlation coefficient ≥ 0.75.

The data were tabulated and expressed as means and standard deviations, percentages or ratios. The significance level for all tests was set at 5%. Statistical analyses were performed using the Statistical Package for the Social Sciences (SPSS) for Windows, version 18.0.1.

### Ethical considerations

This project was approved by the Research Ethics Committee of the Health Sciences Center, UFES (no. 057586/2012), and all participants signed an informed consent statement.

## RESULTS

**Figure 1:**
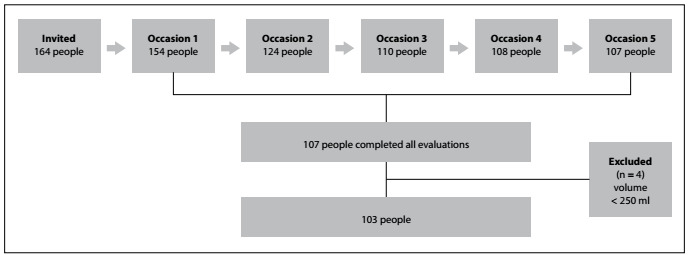
Sample description.

Out of the 164 individuals who were invited to participate, 103 completed the urine collections on all five occasions, as presented in Figure 1. Data from these 103 participants of mean age of 48.3 ± 12 years were analyzed. Individuals with renal disease who were undergoing dialysis, pregnant women and individuals with cognitive limitations were excluded. A total of 154 people fulfilled the first 12-hour urine collection, 124 the second, 110 the third, 108 the fourth and 107 the fifth. Volumes of 12-hour urine collection < 250 ml were excluded from the analysis. After all the exclusions, the final sample for analysis purposes comprised 103 people.

Table 1 shows the characteristics of the sample. The majority were female (64.1%), of socioeconomic class B (53.4%), with higher levels of schooling (80.6%) and white race/color (50.5%). At the first interview, one third of the participants reported adding salt to table preparations (34%), 20.4% reported having a diagnosis of hypertension, 8.7% had diabetes and 55.4% were overweight (overweight or obese).

**Table 1:** Characterization of the sample studied. VALSA Study - Vitória (ES), 2015

Variable	n	%
Sex		
Female	66	64.1
Male	37	35.9
Schooling		
Elementary and high school	20	19.4
Undergraduate and postgraduate university	83	80.6
Race/color		
White	52	50.5
Mixed	51	49.5
Socioeconomic class		
A	30	29.1
B	55	53.4
C	18	17.5
Nutritional status		
Normal	46	44.6
Overweight*	57	55.4
Hypertension diagnosis	21	20.4
Diabetes mellitus diagnosis	9	8.7
Use of salt shaker	35	34

*Overweight = body mass index ≥ 25 kg/m^2^.

Table 2 presents the participants’ averages and standard deviations for sodium intake terciles on the five occasions over the one-year period. The participants in the first tercile presented a mean salt intake that was within the current recommendation (up to 5 g/day), in four out of the five evaluations. In the last tercile, the mean value reached more than three times the recommendation, in four out of the five evaluations.

**Table 2:** Mean and standard deviations (SD) (95% confidence interval) of 12-hour urinary sodium measurements, according to consumption terciles on five occasions over a one-year period. VALSA Study, Vitória (ES), 2015

Occasion	1^**st**^ tercile	2^**nd**^ tercile	3^**rd**^ tercile
	Mean ± SD (95% CI)	Mean ± SD (95% CI)	Mean ± SD (95% CI)
1	5.2 ± 1.4 (2.4-6.8)	8.6 ± 1.1 (6.8-10.3)	12.6 ± 2.1 (10.6-20.3)
2	4.7 ± 1.4 (1.6-6.3)	8.2 ± 0.9 (7.0-10.0)	15.0 ± 5.4 (10.1-31.0)
3	4.6 ± 1.1 (2.1-6.4)	7.9 ± 0.9 (6.4-9.6)	15.4 ± 5.4 (9.9-33.2)
4	3.9 ± 1.3 (0.7-5.5)	7.7 ± 1.0 (5.6-9.5)	15.1 ± 5.4 (9.8-31.2)
5	4.6 ± 1.3 (1.9-6.6)	9.0 ± 1.4 (6.7-11.2)	14.2 ± 2.9 (11.4-27.7)

CI = confidence interval.

Table 3 shows the nighttime 12-hour urinary excretion data regarding sodium and potassium and the estimated salt consumption and energy consumption, and the intake of these same nutrients estimated from the 24-hour food register. Significant differences were observed between the means for urinary excretion of sodium (P = 0.009) and potassium (P < 0.001), but no differences in the values of sodium and energy consumption were observed between the five occasions. The estimates of salt intake in g/day remained constant in both methods, but the mean values found through the 24-hour food register were always lower than the values for salt intake estimated from urinary sodium excretion. A small but significant fluctuation (P = 0.042) in the estimates of potassium intake from the 24-hour food register was found. The only significant difference in relation to the urinary sodium measurement was from the fourth to the fifth occasion. Table 3 also shows that there was satisfactory agreement between the measurements of urinary excretion over the one-year period for both sodium (intraclass correlation coefficient = 0.65; P < 0.001) and potassium (intraclass correlation coefficient = 0.58; P < 0.001), and for estimated salt consumption (intraclass correlation coefficient = 0.64; P < 0.001).

**Table 3:** Mean and standard deviations (SD) of 12-hour nocturnal urinary excretion of sodium and potassium, and estimated salt consumption, on the five occasions evaluated. VALSA Study, Vitória (ES), 2015

Occasion	12-hour urinary excretion			
	Sodium (g/day)	Potassium (g/day)		Estimated salt consumption (g/day)
	Mean ± SD	Mean ± SD		Mean ± SD
1	3.7 ± 1.5	0.9 ± 0.6^#^		9.2 ± 3.6
2	4.3 ± 2.2	1.1 ± 0.60^#^		10.6 ± 5.5
3	3.9 ± 2.0	1.1 ± 0.52		9.9 ± 5.0
4	3.5 ± 1.7^#^	1.0 ± 0.5		8.8 ± 4.3
5	4.3 ± 2.2^#^	1.1 ± 0.6^#^		10.9 ± 5.5
P-value	0.009*	0.001**		0.553**
ICC (P-value)	0.65 (< 0.001)	0.54 (< 0.001)		0.65 (< 0.001)
	**24-hour food registry**			
	**Energy (kcal)**	**Sodium (g/day)**	**Potassium (g/day)**	**Estimated salt consumption (g/day)**
	**Mean ± SD**	**Mean ± SD**	**Mean ± SD**	**Mean ± SD**
1	2228 ± 750	3.4 ± 1.3	3.4 ± 1.8^#^	7.4 ± 2.8
2	2127 ± 818	3.1 ± 1.5	3.2 ± 1.4	6.8 ± 3.2
3	2001 ± 709	3.1 ± 1.5	2.9 ± 1.5^#^	6.7 ± 3.3
4	2197 ± 695	3.4 ± 1.5	3.2 ± 1.6	7.4 ± 3.3
5	2121 ± 723	3.3 ± 1.8	2.9 ± 1.4^#^	7.2 ± 3.9
P-value	0.219*	0.175**	0.042**	0.245**
ICC (P-value)	0,64 (< 0.001)	0.49 (< 0.001)	0.65 (< 0.001)	0.64 (< 0.001)

*Analysis of variance; **Kruskal-Wallis; ^#^difference between means. ICC = intraclass correlation coefficient; SD = standard deviation.

Table 4 shows the correlations between urinary sodium and potassium values and estimated salt consumption obtained on each of the occasions, and shows the averages for the five evaluations. It can be seen that all the correlations were positive and that most of them presented statistical significance. It is important to note that the correlations of the sodium measurements between the first, second and fifth occasions and the averages for the five occasions were moderate (from 0.51 to 0.75), while the correlation between the third occasion and the overall mean was strong (r = 0.76). In relation to potassium, all the correlations were positive, but some were not as significant as those of the fourth occasion in relation to the other measurements. However, a positive and significant correlation was found between the fourth measurement and the mean for the five observations. The measurements of sodium and potassium from the urine collection on the fourth occasion were the ones that differed from the others. A similar result was found in relation to the estimated salt consumption.

**Table 4:** Correlation (r) between measurements of urinary sodium and potassium excretion and estimated salt consumption over a one-year period (five occasions and mean). VALSA Study, Vitória (ES), 2015

Occasion	Occasion				
	1	2	3	4	5
	Sodium				
1	1.000	0.215^*^	0.349^**^	0.190	0.290^**^
2	0.215^*^	1.000	0.396^**^	0.126	0.216^*^
3	0.349^**^	0.396^**^	1.000	0.242^*^	0.464^**^
4	0.190	0.126	0.242^*^	1.000	0.168
5	0.290^**^	0.216^*^	0.464^**^	0.168	1.000
Estimated mean	0.510**	0.617**	0.763**	0.471**	0.712**
**Potassium**					
1	1.000	0.148	0.265^**^	0.172	0.205^*^
2	0.148	1.000	0.384^**^	0.002	0.206^*^
3	0.265^**^	0.384^**^	1.000	0.062	0.306^**^
4	0.172	0.002	0.062	1.000	0.088
5	0.205^*^	0.206^*^	0.306^**^	0.088	1.000
Estimated mean	0.583**	0.586**	0.646**	0.343**	0.566**
**Estimated salt**					
1	1.000	0.215^*^	0.349^**^	0.190	0.290^**^
2	0.215^*^	1.000	0.396^**^	0.126	0.216^*^
3	0.349^**^	0.396^**^	1.000	0.242^*^	0.464^**^
4	0.190	0.126	0.242^**^	1.000	0.168
5	0.290^**^	0.216^*^	0.464^**^	0.168	1.000
Estimated mean	0.510**	0.617**	0.763**	0.471**	0.712**

* P < 0.05; ** P < 0.01. Estimated mean = mean of five occasions.

In addition, analyses were performed to evaluate the correlation between the 24-hour urinary excretion measurements and the 12-hour nocturnal measurements (data not shown in the table). A strong correlation was found between the 24-hour and 12-hour measurements for both sodium and potassium on the first occasion. On the fifth occasion, the strong correlation remained for sodium, while for potassium the correlation was moderate (r = 0.66; P < 0.001).

## DISCUSSION

Satisfactory agreement was found between the five measurements of urinary excretion obtained from a 12-hour period at night, over a one-year period, showing little variability in consumption. Therefore, our data suggest that a single measurement of urinary sodium and potassium provided estimates of reasonable accuracy for the usual mean consumption and may be used in population studies on adults.

Given that the intraclass correlation coefficient is an estimate of the fraction of the total variability of measurements that is due to variations between individuals, it can be inferred that there was relative variability over the one-year period. Nonetheless, it can also be inferred that this did not compromise the use of a single measurement for estimating the usual sodium and potassium intake of the group, because of the level of agreement that was found when using the intraclass correlation coefficient.

The sodium values ​found on the five occasions over the one-year period, both from urinary excretion and from the registry, were higher than the recommended values but lower than those that were ​found in a similar study conducted in the city of Vitória, Espírito Santo, Brazil^12^ and in studies on employees at the same institution as in that study.^13^^,^^14^ This may have been related to the low use of manufactured seasonings, in comparison with natural condiments, which was much cited by the group in the present study.

According to Nakasato,^15^ the main source of sodium in food comes from cooking salt, although this micronutrient is present in almost all foods that have undergone some form of industrial process, such as breads, biscuits and sausages, among others. Significant levels ​of sodium are also found in meats, butter and cheeses. Although the sodium values from the food records of the present study were below those found in urinary excretion, they were probably still elevated, and it has been shown that food records underestimate sodium intake^18^

The sodium values from the urinary excretion and from the registry were transformed into amounts of salt (g). At all times and from both methods, the estimated salt values were above the current recommendation of up to 5 g/day. The estimated intake of salt ascertained through the food register was, on average, 22% lower than the values estimated from urinary excretion. It is likely that this difference arose because the 24-hour food register did not detect addition of salt, or the salt contained in the manufactured seasonings that are used in food preparation.^16^ In food composition tables, the amount of salt in the preparations is defined a priori, and no computation is made regarding further addition of salt or of products that participants in such studies may add during the preparation of foods, such as ready-made seasonings and soy sauce, among others that are rich in sodium.

Sodium intake seemed to be underestimated through the dietary method, and this occurred independently, i.e. without any correlation with the values ​estimated from urinary excretion. This could be expected, because the food register was filled out on the day of urine collection, but not all sodium is eliminated immediately after consumption because urinary excretion depends on a large number of factors, including water balance and osmosis, blood pressure and production of hormones, among others.^17^ Thus, a set of food records compiled over a one-year period can contribute towards clarifying the sources of nutrients and thereby identifying whether excess sodium in the diet is related to higher addition of salt and manufactured seasonings, or whether it comes from processed foods, which cannot be observed by using the biomarker.

Also, as expected, there was a significant correlation between the energy values and the estimates of sodium and potassium intake from the 24-hour food register. Considering that 24-hour food registers are less subject to information bias than are other instruments for estimating food consumption, and that the data were checked by the researchers at the time when the forms were delivered, problems relating to the method used and to the quality of information were minimized in this study.

In a recent study, the sodium values from urinary excretion and from a food frequency questionnaire were similar,^15^ probably due to overestimation of consumption through the dietary method used. In the present study, the mean values for sodium and potassium were lower because the food record evaluated the instantaneous consumption, i.e. what was consumed on the day recorded. Since there were five 24-hour records over the course of the year, the mean from these may have represented the habitual consumption of these nutrients, without the more frequent overestimation observed in food frequency questionnaires.^18^

It is important to emphasize that the sample consisted mostly of women, with a mean age of 48 years, in higher socioeconomic classes and with high schooling levels. These characteristics may determine healthier eating habits.^19^ In addition, the fact that most of them were women meant that their consumption of food was generally lower, which therefore decreased the average consumption of the group. Another issue relates to higher education levels, which may lead to healthier food choices because of greater access to information about nutrition.

The differences found in relation to potassium through the two methods probably related to seasonal consumption of fruits and vegetables, which are important sources of this nutrient.^20^ It is also possible that the lower consumption was already an expected effect from some actions taken by manufacturers with the aim of reducing the sodium levels in processed foods. However, it is more likely that the individuals added less salt to the food during the preparation and used less industrialized seasonings.

## CONCLUSION

Measurement of 12 hours of nocturnal urinary excretion of sodium and potassium can be used to evaluate habitual consumption of these nutrients in population studies, since good agreement among the five measurements evaluated over a one-year period was observed. However, it is important to take into account other information, especially factors relating to dietary habits and behavior and the types of food consumed, in relation to these nutrients.

The biological marker, in this case, is the one that best reproduced the consumption of these nutrients, but it is not able to identify the amounts of sodium/salt added to the food. Thus, the single 12-hour urinary excretion measurement can be used to evaluate changes to the population’s behavior regarding sodium and potassium intake, and it may contribute towards monitoring of governmental actions aimed at reducing sodium intake and, consequently, improving health conditions and the cardiovascular morbidity and mortality profile.
